# A meta-research review of the use of flexible meta-regression in biomedical systematic reviews published between 2011 and 2021

**DOI:** 10.1017/rsm.2025.10067

**Published:** 2026-06-04

**Authors:** Marc Parsons, Jingjun (Victor) Chen, Andrea Benedetti, Russell Steele

**Affiliations:** 1 Department of Epidemiology, Biostatistics, and Occupational Health, McGill University, Canada; 2 Mathematics and Statistics, McGill University, Canada

**Keywords:** flexible regression, fractional polynomials, meta-regression, splines

## Abstract

Flexible meta-regression is an important facet of biomedical research. These methods employ basis expansions to model the relationship between exposure and outcome. Examples include splines, fractional polynomial, and local regression models. The extent to which flexible meta-regression has been appropriately employed and reported in the past has not been studied. This study aims to provide an overview of flexible meta-regression for researchers as well as summarize the practical use and reporting of these methods in the epidemiological literature. A systematic literature search of EMBASE, MEDLINE, and the Cochrane Library identified systematic reviews published between 2011 and 2021 which employed flexible meta-regression methods. Data on study characteristics were extracted and validated by two independent researchers. Given the lack of guidance on reporting standards for flexible meta-regression, we proposed a novel 9-item checklist. This was used to assess reporting quality in included studies. A total of N=346 eligible studies were identified and N=86 were randomly sampled for full data extraction and analysis. The number of reviews using flexible regression methods increased over time. Spline models were the most frequently employed class of methods. There were no apparent trends in the use of the three methods over country of affiliation, number of authors, or clinical context. In over two thirds of reviews sampled, methods were found to have not been reported consistently. Further work is needed to improve the reporting of these methods to ensure research transparency and reproducibility.

## Highlights

### What is already known?

- Flexible meta-regression can be used to model outcome–exposure relationships in meta-analyses. Different basis expansions of the exposure variable can be used in flexible meta-regression, including splines and fractional polynomials.
- Reporting guidelines in meta-analysis are important for ensuring transparency and reproducibility of research. However, no such guidelines exist for the reporting of studies employing flexible meta-regression.

### What is new?

- The number of published studies employing flexible meta-regression increased dramatically over the period 2011–2021.
- Among the classes of basis expansions considered, spline models were more commonly used than the other methods.
- Reporting quality was generally poor among included studies, regardless of the method used.

### Potential impact for RSM readers

- Our article provides an overview of the use of flexible meta-regression for researchers.
- Researchers may consult our list of reporting guidelines to improve the transparency and reproducibility of their flexible meta-regression analyses.

## 1 Introduction

Systematic reviews and meta-analyses are a cornerstone of epidemiological research. These studies synthesize the results of published literature on a given medical topic, providing the notable benefit of increased statistical power over any single study. Methods which meta-analyze non-linear exposure–outcome trajectories are called flexible meta-regression methods.

Flexible meta-regression proceeds in a similar way to standard meta-regression, with the main difference being replacing linear exposure term *x* with the terms of a basis expansion $B=(f_{1}(x),f_{2}(x),\ldots ,f_{m}(x))$ in order to flexibly model the trajectory over outcome *y* over its range. Many such bases have been used in practice, including: polynomial regression, where *x* is transformed into a polynomial expansion of degree *m* ^1^; fractional polynomial (FP) models, where a variable selection algorithm pulled from a set of power transformations of *x* chooses the best fitting terms for the basis^2^ ^–^ ^4^; and splines, which divide the range of *x* based on pre-specified knots and constructs low-order polynomials between them.^5^ ^–^ ^7^ See Appendix A for an overview of these methods.

Transparent reporting of methods is important to ensure scientific reproducibility. To our knowledge, no comprehensive overview of flexible meta-regression methods has been published to date. Past reviews on other meta-regression methods and standard flexible (non-meta-)regression have identified reporting issues in the published literature.^5^ ^,^ ^8^ ^–^ ^11^

We conducted a systematic search of the use of flexible meta-regression methods from 2011 to 2021 to describe: (a) their use and (b) the quality of their reporting.

## 2 Methods

### 2.1 Literature search

We searched EMBASE and MEDLINE for studies employing flexible meta-regression methods published between 2011 and 2021. Search terms were adapted from a pre-defined search filter to identify systematic reviews^12^ (Appendix B). The Cochrane Collaboration Library was also searched using the search terms related to flexible meta-regression in the title and abstract fields (lines 24–33). Included abstracts and studies not published in English or French were translated using professional translators.

### 2.2 Inclusion and exclusion criteria

Two independent reviewers screened the titles and abstracts of identified studies at the first stage. Inclusion at this stage was solely based on whether the study was a biomedical systematic review. Any studies included by at least one of the reviewers were moved forward to the second stage of study selection. In the second stage, the full text was screened for inclusion by two independent reviewers. As the Cochrane Collaboration Library contains only meta-studies by design, results from this database were immediately moved forward to the second stage. Exclusion criteria at the second stage are listed in Appendix C. Any discrepancies between the two independent assessors were first discussed between them to achieve consensus. Discrepancies which were not resolved through discussion were brought to a third reviewer for final consensus. Inclusion/exclusion of identified studies was conducted in the DistillerSR systematic review software^13^ and Microsoft Excel.^14^

### 2.3 Data extraction and validation

Due to the large number of included records, a decision was made to conduct full data extraction and validation on a random subsample of studies satisfying inclusion criteria as is standard practice in reviews including a large number of studies.^15^ Given the substantial sample of included studies, the time required to extract data, and the methodological scope of this review, it was determined that a sample of 25% was sufficient for full data extraction. A researcher extracted data from the included studies. These data were then validated by a second independent researcher. The variables to be extracted were determined *a priori* based on the research objectives. Standard publication characteristics were extracted (author country affiliations, number of authors, journal of publication, clinical field of study, and journal impact factor [JIF]). The primary clinical field of study was coded from the following categories: nutrition, public health, cardiology, nephrology, oncology, psychology, and other. The type of meta-analysis (MA or IPDMA as well), whether network MA (NMA) methods were used, and the number of included studies were also extracted. JIF in the publication year was obtained from Clarivate’s Journal Citations Reports online tool. If JIF was not available, the value for the nearest year was taken, with a preference for an earlier rather than later year. Publication year as well as the number of authors were obtained directly from bibliographic data. These were the only characteristics available for the entire sample, obtained directly from the bibliographic data of the initial search. All other publication characteristics were available only for the subsample from which further data were extracted. As the assessment of the risk of epidemiological bias is beyond the scope of this methodological study, included studies were not assessed for risk of bias (RoB).

The flexible meta-regression method(s) employed were extracted verbatim in a free text field. These were then coded into one of four categories: splines; local regression (LR); FP; and other methods, such as moving averages or (non-fractional) polynomial regression.

### 2.4 Proposed reporting guidelines

Given the lack of specific guidance for the reporting of flexible meta-regression methods, we propose a set of reporting items (Table 1). These were developed with knowledge of the methodological literature on flexible meta-regression as well as an initial overview of the included studies in the present review. Typically, reporting quality in a meta-study is measured through a checklist, such as the Preferred Reporting Items of Systematic reviews and Meta-Analyses (PRISMA).^16^ These guidelines encompass all aspects of the systematic review process. The reporting guidelines presented here are focused on the statistical methods employed in meta-studies using flexible meta-regression methods. The PRISMA statements concerning individual participant data MA (IPDMA)^17^ and NMA^18^ provide examples of detailed guidance on statistical methodology, in particular. We referred to Perperoglou et al.^5^ for recommendations regarding spline models.

**Table 1:** Proposed reporting guidelines for flexible meta-regression methods in systematic reviews Table 1 Long description.

Reporting item	Item description
	Did the study…
1	present any results of the flexible meta-regression method employed?
2	include a statistical methods section?
3	report which flexible meta-regression methods were used?
4	include a citation for the flexible meta-regression methods used?
5	report which statistical tests were used, *if applicable*?
6	clearly state the flexible meta-regression method by name?
7	report flexible meta-regression method specifically in the methods section?
8	report details around number and placement of spline knots, *if applicable*?
9	report flexible meta-regression method in included figure, *if applicable*?

We employed our proposed reporting guidelines to extract information on the quality of reporting of flexible meta-regression methods from subsampled studies. Each of the 9 included items presents as a yes or no question, with a positive answer indicating good reporting and a negative one indicating the contrary. Items 5, 8, and 9 were only considered for studies which claimed to either have conducted a statistical test in their flexible meta-regression (Item 5), used spline methods (Item 8), or produced a figure of flexible meta-regression results (Item 9), respectively.

### 2.5 Data analysis

Descriptive statistics (*n* and % of studies) were calculated for important study characteristics. For numerical publication factors (impact factor, number of authors, and number of included studies), sample means and standard errors were also calculated. ANOVA was used to compare between group means of numerical variables. Chi-squared tests were used to compare group proportions. As the full sample is the target population of this review, we correspondingly used the fixed-population correction

$$\begin{array}{r} \text{FPC}=\sqrt{\frac{N_{\text{total}}-N_{\text{samp}}}{N_{\text{total}}-1}}=\sqrt{\frac{346-86}{346-1}}\approx 0.868 \end{array}$$

to adjust standard errors (SEs) estimated from the subsample, where $N_{\text{total}}=346$ is the number of studies in the target population and $N_{\text{samp}}=86$ is the number of studies sampled without replacement from the population.^19^ The subsample was compared to the full sample by the number of authors and publication year, the two bibliographic variables which the samples shared. Natural spline models with k=3 equally-spaced knots were used to describe trends in scatterplots. All analyses were conducted in R.^20^

### 2.6 Literature search update

After conducting the initial search, it was determined that studies using FP methods may not have been adequately captured. It was decided that an update would be conducted with an edited search strategy. In the new search, line 33 was modified to include the term “fractional poly*” (see Appendix B). The results of this search were then filtered using an anti-join to obtain the unique updated results. These were then assessed for eligibility in the present review. A random subsample of 25% of the eligible studies—the same procedure for the initial search—was included in data extraction, validation, and analysis.

## 3 Results

### 3.1 Search results

**Figure 1:**
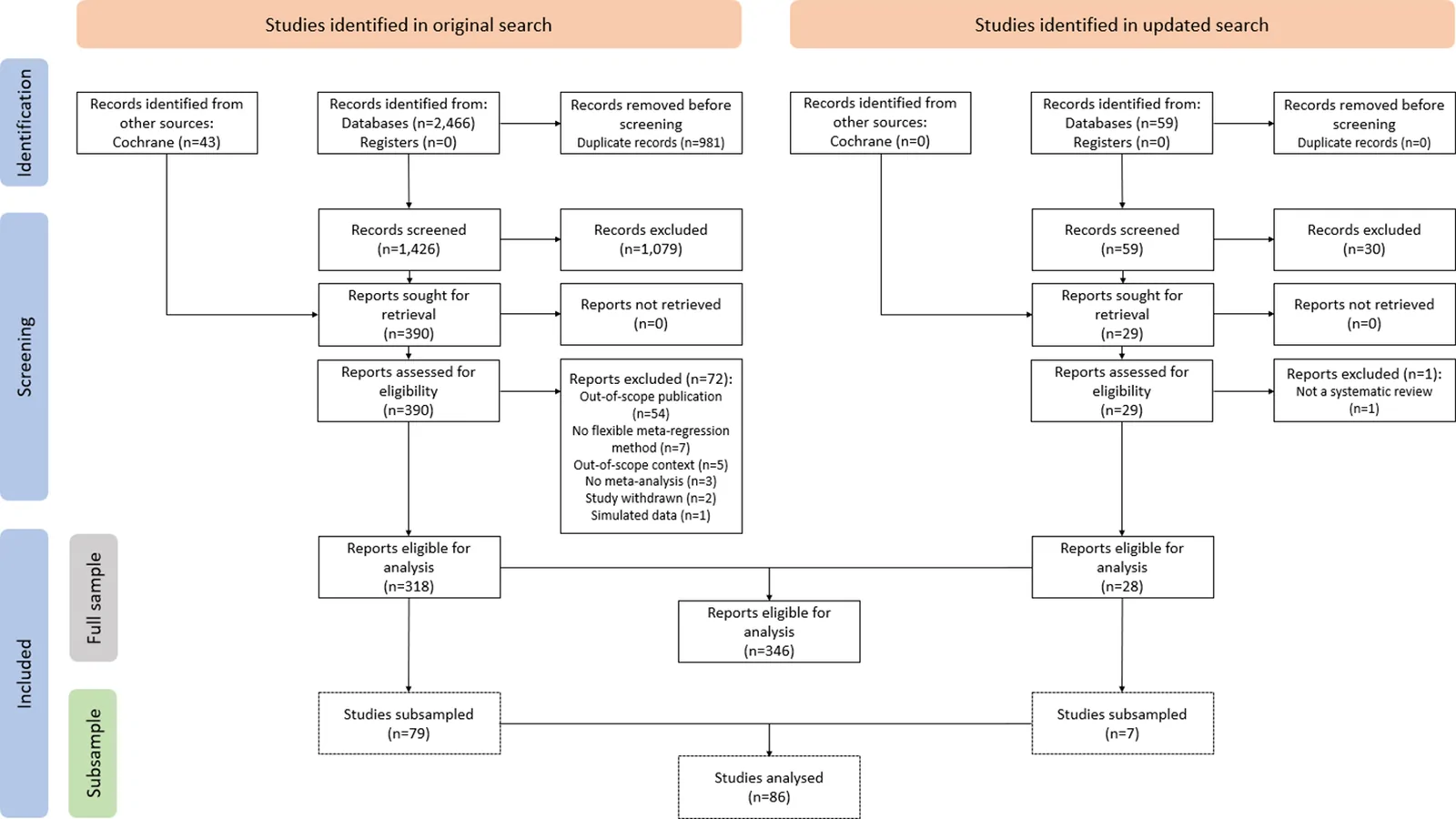
PRISMA flowchart outlining initial search, updated search, and screening results. *Note*: Studies identified in the updated search as described in the methods section in order to capture fractional polynomial models. As described in Appendix B, the results were anti-joined to the results of the original search in order to capture studies which were not initially identified. Figure 1 Long description.

Our initial search identified a total of N=2,466 records, of which N=981 were duplicates (Figure 1). The remaining N=1,485 records screened included an additional N=59 studies identified in the search update. We excluded N=1,109 studies at the first stage of inclusion. We retrieved full-texts for all N=419 included studies as well as N=43 identified from the Cochrane database. Of these, a total of N=73 were excluded at the second stage, leaving N=346 studies meeting inclusion criteria. A random subsample of 25% ( N=86 ) was selected for full extraction.

To assess the representativeness of the subsample, we compared it to the full sample using the bibliographic data available in both datasets. The subsample did not statistically significantly differ from the full sample in either the mean number of authors ( 6.9 , SD =4.6 , and 7.3 , SD =6.9 , respectively) nor in the distribution of studies over publication year.

### 3.2 Use of flexible meta-regression methods

**Figure 2:**
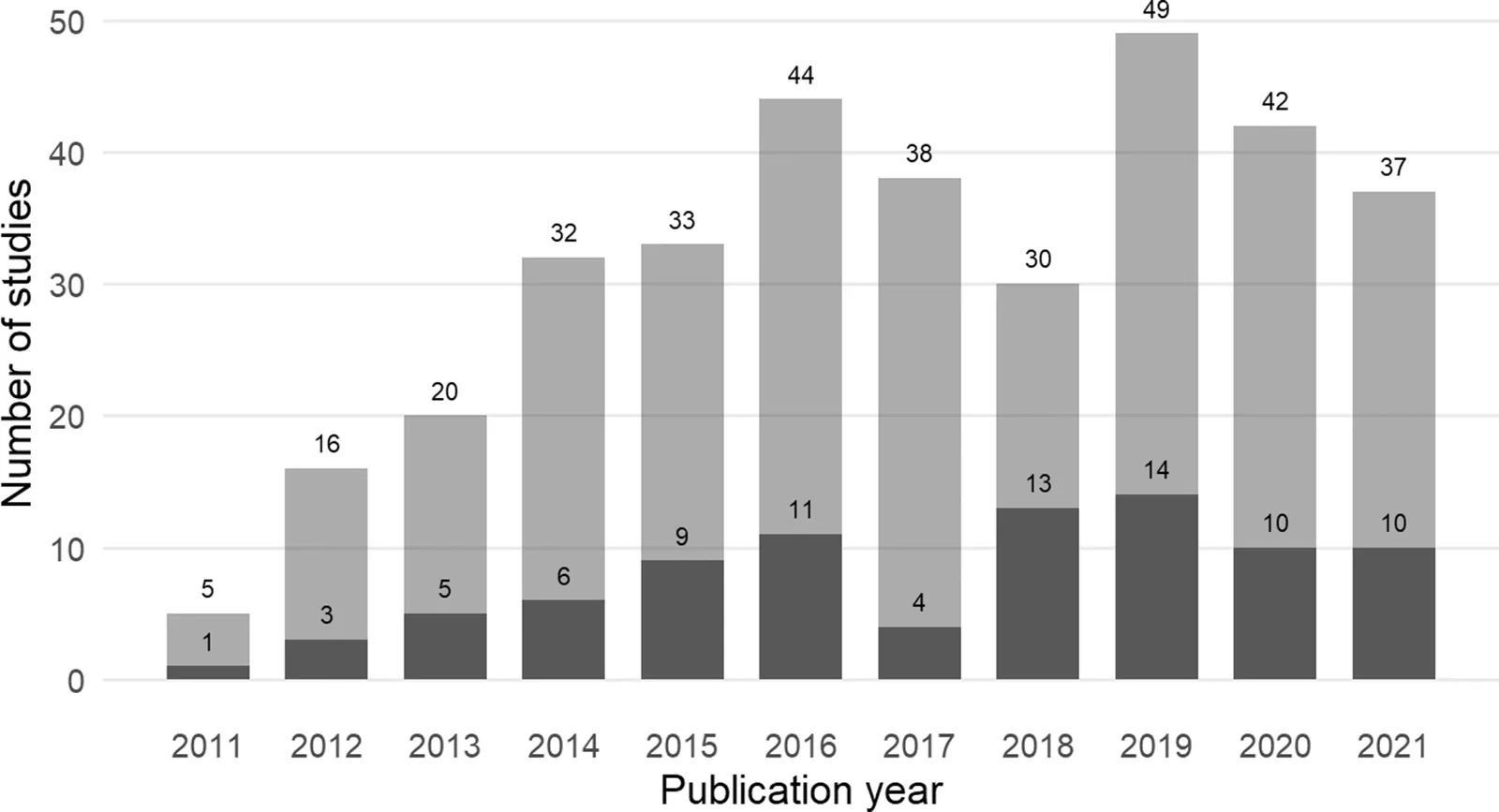
Histogram of identified and sub-sampled studies by publication year. *Note*: The lightly shaded bars show the frequency by publication year of the N=346 included studies. The dark bars show the same frequency but for the N=86 sub-sampled studies from which additional data were extracted. Figure 2 Long description.

The number of studies using flexible meta-regression methods increased over 2011–2021 (Figure 2, p<0.001 ). Of the N=347 studies, only N=5 ( 1.4% ) were published in 2011. The yearly frequency of publications increased eight-fold between 2011 and 2016 ( N=44 , 12.7% ). Between 2016 and 2021, numbers remained constant, with a peak in 2019 of N=49 ( 14.1% ).

Of the N=86 for which additional data were extracted, spline models were the most widely used method ( N=67 , 77.9% ). This was statistically significant ( p<0.001 ). FP and LR were less common among included studies ( N=17 , 19.8% , and N=5 , 5.8% , respectively). Two studies ( 2.3% ) used other methods (a moving average model and a piecewise linear model) while two reported using flexible meta-regression but the particular method was unclear. Excluding the two studies with unclear methods, N=77 ( 91.7% ) employed a single method while N=6 ( 7.1% ) employed two. Four ( 4.8% ) used both spline and FP. Two studies ( 2.4% ) using other methods also employed a second: one of these also used splines and the other used LR. While FP models were more commonly used toward the end of the study period, no statistically significant trend was observed between the flexible meta-regression methods and year of publication.

**Figure 3:**
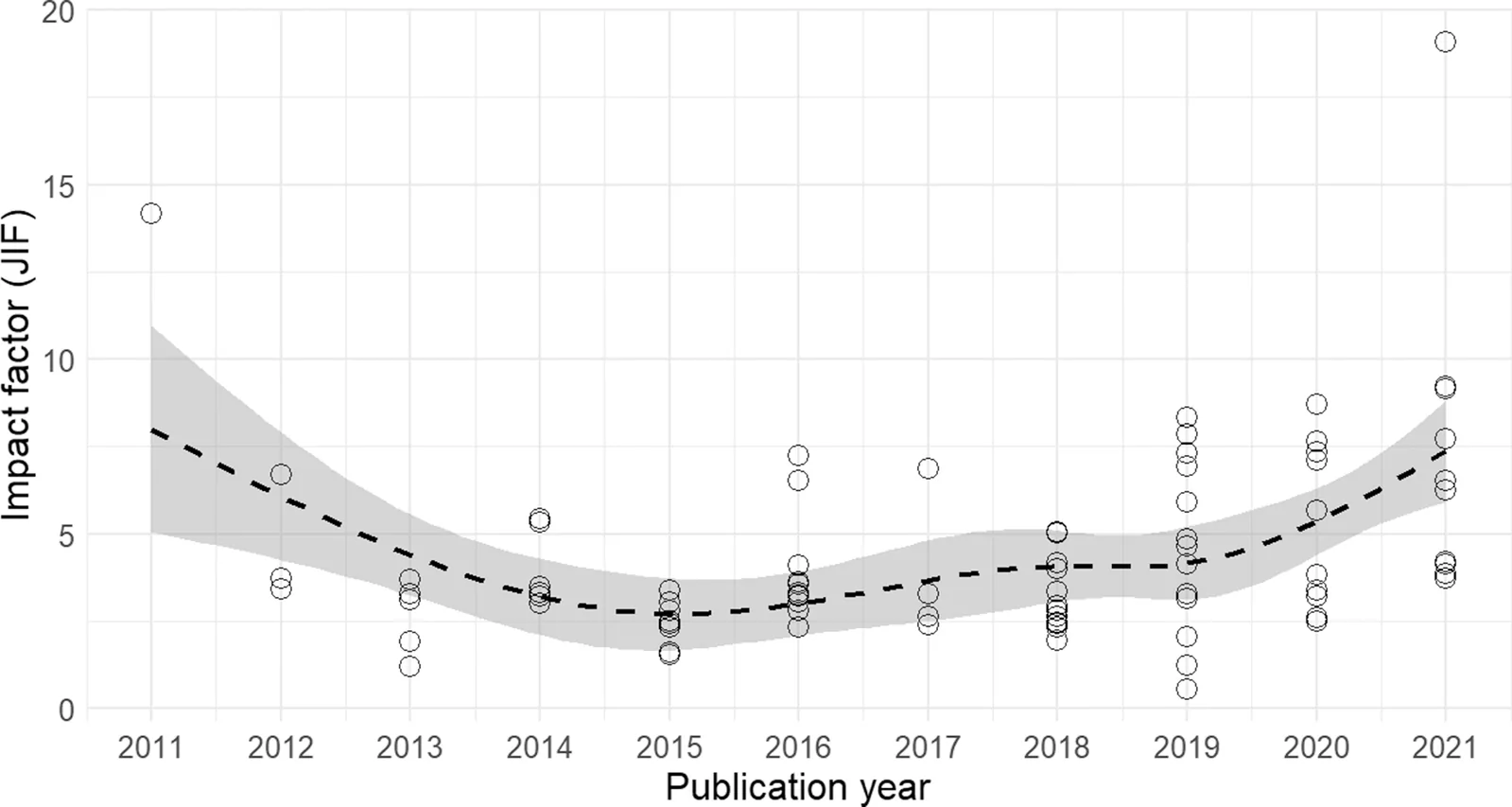
Scatterplot of impact factor over publication year of sub-sampled studies. *Note*: The dashed line is the fitted estimate for mean impact factor over time obtained through a linear regression model using a natural cubic spline basis with k=3 equally-spaced knots over the range of publication year. The 95% confidence ribbon (shaded gray) was obtained using the fitted SE of the spline regression model. Figure 3 Long description.

The most common clinical field was nutrition ( N=37 ) followed by public health ( N=24 ) and cardiology ( N=9 ). Mean JIF was 4.4 (SD =4.7 ). Studies had a mean of 7.5 (SD =4.0 ) authors and of 22.8 (SD =18.2 ) included studies. Only two studies were IPDMAs. Five countries had five or more publications with at least one affiliated author: China ( N=48 , 55.8% ), the USA ( N=16 , 18.6% ), the UK ( N=11 , 12.8% ), Iran ( N=9 , 10.5% ), and Italy ( N=5 , 5.8% ). No studies used NMA methods (not shown in Table 2). Mean JIF varied slightly over the study period (Figure 3).

**Table 2:** Summary statistics of sub-sampled studies by method and publication characteristic Table 2 Long description.

							Unique studies*
Publication	characteristic	Flexible meta-regression method (*n*)					( N=86 )
		Spline	FP	LR	Other	Unclear	
Flexible meta-	Spline	67	4	0	1	0	67
regression	FP	4	17	0	0	0	17
method	LR	0	0	5	1	1	5
	Other	1	0	1	2	0	2
	Unclear	0	0	1	0	2	2
Publication	2011–2013	7	2	0	0	0	9
year	2014–2015	10	3	3	1	1	15
	2016–2017	14	2	0	0	0	15
	2018–2019	23	2	1	0	1	27
	2020–2021	13	8	1	1	0	20
Clinical field	Nutrition	30	9	0	0	0	37
	Public health	19	3	2	2	0	24
	Cardiology	7	1	0	0	1	9
	Oncology	5	2	1	0	0	7
	Nephrology	5	1	0	0	0	5
	Other	1	1	1	0	1	3
	Psychology	0	0	1	0	0	1
Impact factor	Low	20	5	4	0	2	29
(JIF)**	Mid	26	3	0	2	0	29
	High	21	9	1	0	0	28
	*mean (SD)*	4.4 (2.9)	5.0 (2.5)	3.4 (2.6)	-	-	4.4 (4.7)
No. of authors	1–3	13	2	1	0	0	16
	4–6	21	6	1	0	1	28
	7–10	19	6	3	1	1	26
	> 10	14	3	0	1	0	16
	*mean (SD)*	7.4 (4.7)	7.4 (4.2)	5.8 (2.4)	-	-	7.5 (4.0)
No. of studies	2–10	22	3	0	0	0	25
	11–20	22	8	1	1	0	28
	21–30	9	3	1	0	0	13
	> 30	14	3	3	1	2	20
	*mean (SD)*	21.5 (19.0)	19.8 (11.9)	35.2 (14.6)	-	-	22.8 (18.2)
Type of MA	Standard MA	66	16	5	2	2	84
	IPDMA	1	1	0	0	0	2
Country***	Argentina	0	0	1	1	0	1
	Australia	2	2	0	0	0	4
	Brazil	0	2	0	0	0	2
	Canada	1	3	0	0	0	4
	China	43	6	2	1	0	48
	Finland	1	0	0	0	0	1
	France	0	1	0	0	0	1
	Germany	2	2	0	0	0	4
	Ghana	1	0	0	0	0	1
	Greece	0	0	2	0	1	2
	Iran	3	6	0	0	0	9
	Italy	5	0	0	0	0	5
	Japan	4	0	0	0	0	4
	Korea	1	0	0	0	0	1
	Netherlands	1	0	0	0	1	2
	Norway	2	1	0	0	0	2
	Poland	3	0	0	0	0	3
	Spain	1	0	0	0	0	1
	Sweden	3	0	0	0	0	3
	Switzerland	0	1	0	0	0	1
	UK	6	4	1	0	2	11
	USA	12	3	2	1	0	16
	Vietnam	0	1	0	0	0	1

* Individual studies may use more than one method.

** Categories based on JIF tertiles. Low: < 3.0; Mid: 3.0–4.2; High: > 4.2.

*** Country/countries of affiliation of study authors as listed in the publication. Authors may have more than one country of affiliation. FP = fractional polynomial models; IPDMA = individual participant data meta-analysis; JIF = journal impact factor; LR = local regression; MA = meta-analysis.

**Figure 4:**
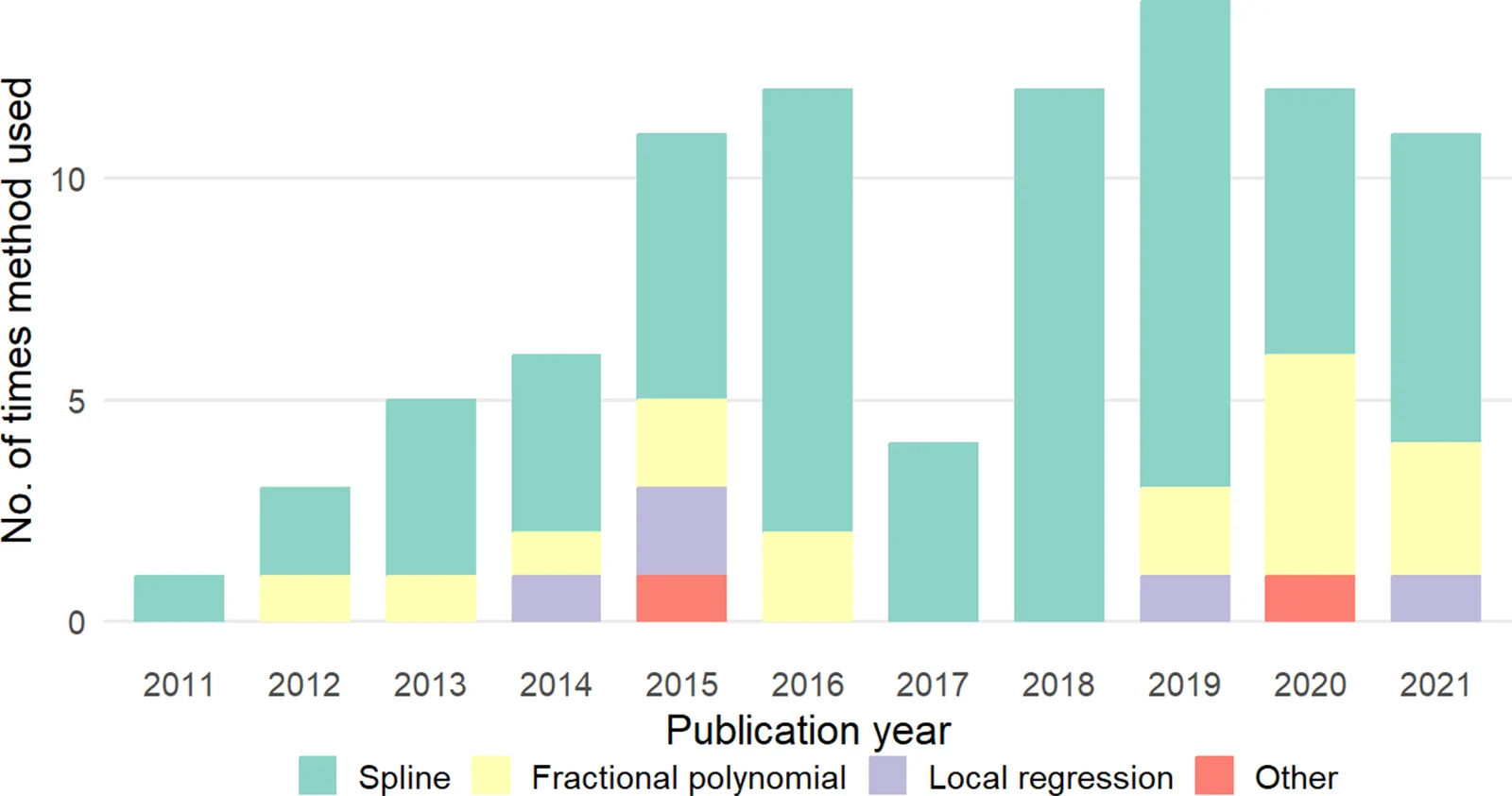
Histogram of sub-sampled studies by publication year and flexible meta-regression method. Figure 4 Long description.

**Figure 5:**
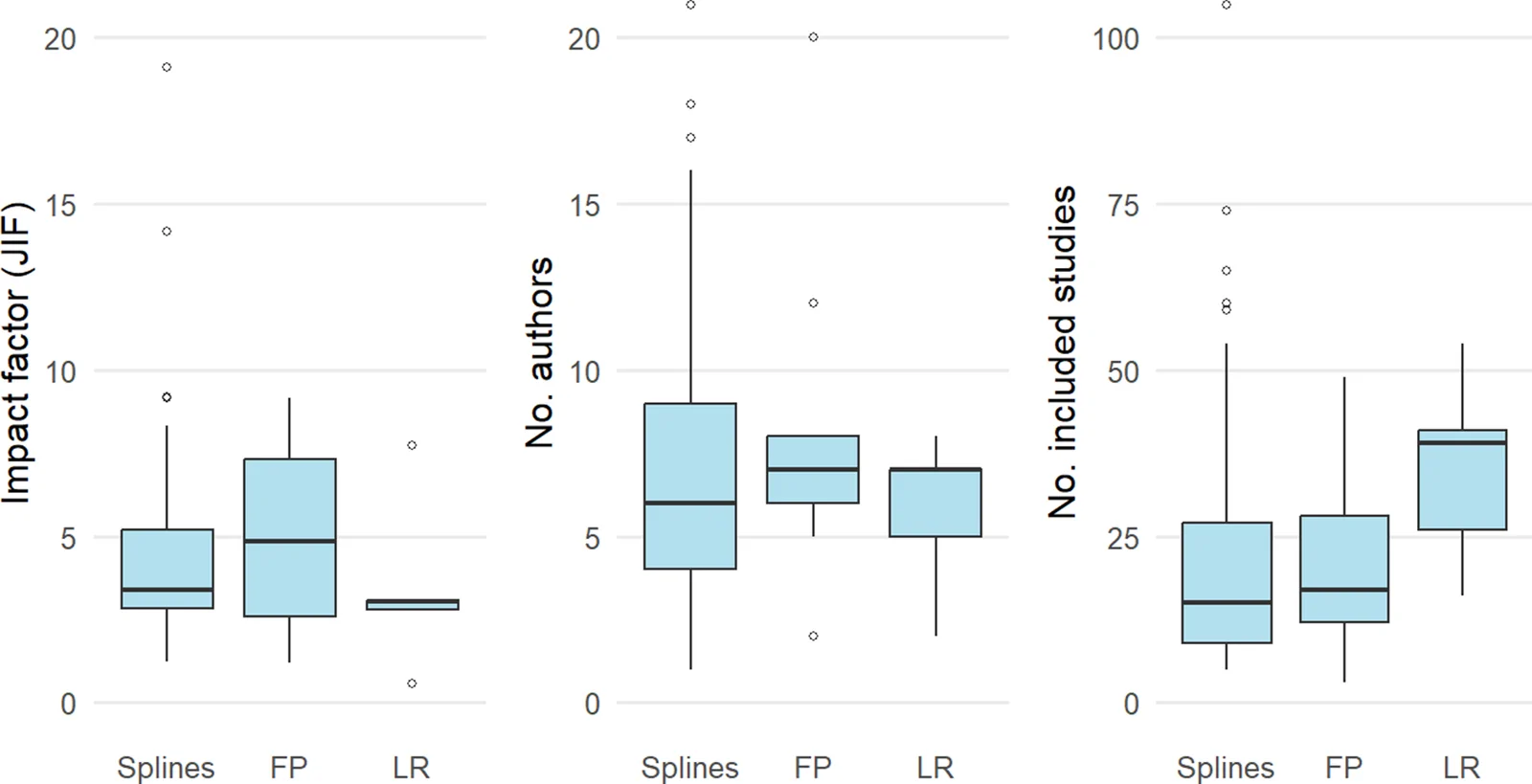
Boxplot of impact factor, number of authors, and number of included studies by flexible meta-regression method. *Note*: Due to small sample sizes in the other categories, only the results for spline ( N=67 ), FP ( N=17 ), and LR ( N=5 ) models are shown here. Studies may use more than one method. FP = fractional polynomial; LR = local regression. Figure 5 Long description.

Descriptively, there were differences in certain publication characteristics between the methods used (Table 2). FP models became more common over time relative to other models (Figure 4). However, of the methods considered in this article, splines were the sole method used each year and were most commonly used in every single year. JIF differed somewhat between methods. Over half of studies using FP were published in the highest JIF tertile journals (JIF >4.2 ) compared to around a third for splines. The mean JIF of papers using FP ( 5.0 , SD =2.5 ) was higher than that of those using splines ( 4.4 , SD =2.9 ) or LR ( 3.4 , SD =2.6 ) (Table 2 and Figure 5). The mean number of included studies was highest for those using LR ( 35.2 , SD =14.6 ) compared to spline ( 21.5 , IQR =19.0 ) or FP ( 19.8 , IQR =11.9 ) methods. However, the five reviews, including the largest number of studies, all employed spline methods. None of the tests for differences between the three methods presented here were significant at the 95% confidence level, however.

### 3.3 Quality of reporting

Overall, 67.4% of studies ( N=58 ) did not meet at least one of the reporting items (Table 3). The item with the worst reporting was Item 9 (reporting the method in included figures, *if applicable*). 44.4% ( N=32 ) of the N=72 studies, including a figure, did not meet this item. Item 1 (does the study present any results of the flexible meta-regression method employed) had the best reporting, with only one study not meeting it.

**Table 3:** Number of sub-sampled studies by reporting item issue and publication characteristics Table 3 Long description.

	Reporting item, *n* (%)										Any of 1–9
		1	2	3	4	5	6	7	8	9	
	Does not meet item	1 (1.2)	2 (2.3)	8 (9.3)	19 (22.1)	18 (26.9)	2 (2.3)	9 (10.5)	13 (19.4)	32 (44.4)	58 (67.4)
	Total applicable	86	86	86	86	67	86	86	67	72	86
Flexible meta-	Spline	1 (1.5)	0 (0.0)	4 (6.0)	8 (11.9)	14 (24.6)	0 (0.0)	4 (6.0)	13 (19.4)	21 (39.6)	40 (59.7)
regression	Fractional polynomial	0 (0.0)	1 (5.9)	3 (17.6)	6 (35.3)	5 (41.7)	0 (0.0)	5 (29.4)	-	8 (57.1)	14 (82.4)
method*	Local regression	0 (0.0)	0 (0.0)	0 (0.0)	4 (80.0)	-	0 (0.0)	0 (0.0)	-	3 (60.0)	5 (100.0)
	Other	0 (0.0)	0 (0.0)	1 (50.0)	0 (0.0)	0 (0.0)	0 (0.0)	0 (0.0)	-	-	1 (50.0)
	Unclear	0 (0.0)	1 (50.0)	1 (50.0)	2 (100.0)	1 (100.0)	2 (100.0)	0 (0.0)	-	-	2 (100.0)
Publication	2011–2013	0 (0.0)	0 (0.0)	1 (11.1)	2 (22.2)	2 (28.6)	0 (0.0)	0 (0.0)	1 (14.3)	5 (62.5)	7 (77.8)
year	2014–2015	0 (0.0)	0 (0.0)	0 (0.0)	3 (20.0)	1 (9.1)	1 (6.7)	1 (6.7)	0 (0.0)	5 (38.5)	8 (53.3)
	2016–2017	1 (6.7)	0 (0.0)	0 (0.0)	1 (6.7)	2 (14.3)	0 (0.0)	1 (6.7)	1 (7.1)	4 (28.6)	7 (46.7)
	2018–2019	0 (0.0)	1 (3.7)	2 (7.4)	6 (22.2)	5 (25.0)	1 (3.7)	2 (7.4)	5 (21.7)	8 (36.4)	16 (59.3)
	2020–2021	0 (0.0)	1 (5.0)	5 (25.0)	7 (35.0)	8 (53.3)	0 (0.0)	5 (25.0)	6 (46.2)	10 (66.7)	20 (100.0)
Impact factor	Low	0 (0.0)	1 (3.4)	2 (6.9)	6 (20.7)	5 (25.0)	2 (6.9)	2 (6.9)	2 (10.0)	9 (36.0)	18 (62.1)
(JIF)**	Mid	0 (0.0)	1 (3.4)	4 (13.8)	5 (17.2)	7 (25.9)	0 (0.0)	3 (10.3)	3 (11.5)	13 (52.0)	19 (65.5)
	High	1 (3.6)	0 (0.0)	2 (7.1)	8 (28.6)	6 (30.0)	0 (0.0)	4 (14.3)	8 (38.1)	10 (45.5)	21 (75.0)
Clinical field	Nutrition	1 (2.7)	1 (2.7)	4 (10.8)	6 (16.2)	8 (24.2)	0 (0.0)	6 (16.2)	5 (16.7)	18 (58.1)	24 (64.9)
	Public health	0 (0.0)	0 (0.0)	3 (12.5)	4 (16.7)	4 (25.0)	0 (0.0)	2 (8.3)	5 (26.3)	9 (45.0)	16 (66.7)
	Cardiology	0 (0.0)	1 (11.1)	1 (11.1)	3 (33.3)	3 (37.5)	1 (11.1)	0 (0.0)	1 (14.3)	3 (37.5)	7 (77.8)
	Nephrology	0 (0.0)	0 (0.0)	0 (0.0)	1 (20.0)	1 (25.0)	0 (0.0)	1 (20.0)	0 (0.0)	0 (0.0)	3 (60.0)
	Oncology	0 (0.0)	0 (0.0)	0 (0.0)	3 (42.9)	2 (50.0)	0 (0.0)	0 (0.0)	2 (40.0)	1 (20.0)	6 (85.7)
	Psychology	0 (0.0)	0 (0.0)	0 (0.0)	1 (100.0)	-	0 (0.0)	0 (0.0)	-	1 (100.0)	1 (100.0)
	Other	0 (0.0)	0 (0.0)	0 (0.0)	1 (33.3)	0 (0.0)	1 (33.3)	0 (0.0)	0 (0.0)	0 (0.0)	1 (33.3)
	Does not meet item	1 (1.2)	2 (2.3)	8 (9.3)	19 (22.1)	18 (26.9)	2 (2.3)	9 (10.5)	13 (19.4)	32 (44.4)	58 (67.4)
	Total applicable	86	86	86	86	67	86	86	67	72	86
No. of authors	1–3	0 (0.0)	0 (0.0)	0 (0.0)	3 (18.8)	2 (16.7)	0 (0.0)	1 (6.2)	2 (15.4)	3 (21.4)	7 (43.8)
	4–6	0 (0.0)	0 (0.0)	2 (7.1)	6 (21.4)	4 (17.4)	1 (3.6)	1 (3.6)	4 (19.0)	14 (58.3)	20 (71.4)
	7–9	1 (3.8)	2 (7.7)	3 (11.5)	8 (30.8)	8 (42.1)	1 (3.8)	7 (26.9)	5 (26.3)	9 (47.4)	19 (73.1)
	> 10	0 (0.0)	0 (0.0)	3 (18.8)	2 (12.5)	4 (30.8)	0 (0.0)	0 (0.0)	2 (14.3)	6 (40.0)	12 (75.0)
No. of studies	2–10	0 (0.0)	0 (0.0)	0 (0.0)	2 (8.0)	6 (27.3)	0 (0.0)	2 (8.0)	4 (18.2)	11 (57.9)	16 (64.0)
	11–20	0 (0.0)	1 (3.6)	4 (14.3)	5 (17.9)	8 (33.3)	0 (0.0)	5 (17.9)	3 (13.6)	8 (33.3)	19 (67.9)
	21–30	0 (0.0)	0 (0.0)	1 (7.7)	3 (23.1)	2 (20.0)	0 (0.0)	2 (15.4)	1 (11.1)	5 (41.7)	7 (53.8)
	> 30	1 (5.0)	1 (5.0)	3 (15.0)	9 (45.0)	2 (18.2)	2 (10.0)	0 (0.0)	5 (35.7)	8 (47.1)	16 (80.0)
Type of MA	Standard MA	1 (1.2)	2 (2.4)	8 (9.5)	18 (21.4)	18 (27.3)	2 (2.4)	9 (10.7)	12 (18.2)	32 (45.1)	57 (67.9)
	IPDMA	0 (0.0)	0 (0.0)	0 (0.0)	1 (50.0)	0 (0.0)	0 (0.0)	0 (0.0)	1 (100.0)	0 (0.0)	1 (50.0)

*Note*: Reporting items are outlined in full in Table 1. Note that Items 5, 8, and 9 are not applicable to all studies.

* Reporting items are assessed by *method* and not by *study*. Individual studies may use more than one method, this may mean that a study which uses two separate methods may meet the guidelines for one method but not the other, for example.

** Categories based on JIF tertiles. Low: < 3.0; Mid: 3.0–4.2; High: > 4.2. FP = fractional polynomial models; IPDMA = individual participant data meta-analysis; JIF = journal impact factor; LR = local regression; MA = meta-analysis.

Studies using LR ( N=5 ) or with unclear methods ( N=2 ) all had at least one item which was not met. Spline studies were moderately less likely to have at least one unmet reporting item ( N=30 , 59.7%) than FP ( N=14 , 82.4%) methods, although this difference was not statistically significant. In general, there was no statistically significant trend over the considered methods in reporting quality, defined as not meeting at least one checklist item.

Reporting was better in studies published midway through 2011–2021 (Table 3 and Figure 3). All studies from 2020 to 2021 ( N=20 ) did not meet at least one of the items. Overall, studies from the highest JIF tertile journals showed worse reporting (75.0% not meeting at least one item) than those published in the lower tertiles (62.1% and 65.5% for the lowest and middle tertiles, respectively). Again, however, this finding was not statistically significant. Of the top five observed clinical fields in the subsample for which data were extracted, reporting was best in nephrology while it was worst in cardiology. Studies with 1–3 authors had markedly better reporting (43.8% not meeting at least one item) than those with four or more (between more than 70.0%).

## 4 Discussion

We identified N=346 systematic reviews published from 2011 to 2021 which employed flexible meta-regression methods. Over this period, the number of studies increased. This corresponds with previous research showing the increasing popularity meta-regression.^21^ A 2019 study found that the number of published meta-analyses increased by over 4,000% between 1995 and 2017.^22^ Similarly, Tipton et al.^23^ found that the number of meta-regression studies (rather than other meta-analyses) increased dramatically since the mid-1970s. They found that the number of studies including “meta-analysis” and “meta-regression” or “meta regression” in the title increased from N=1 in 1980–1989 to N=408 in 2010–2017. Our review identified N=188 studies which employed flexible meta-regression in 2011–2017. While the search strategies between the two studies differed, this suggests that around half of published meta-regression studies employed flexible methods in this period.

Splines were employed more frequently in the included studies than the other considered models. This persisted over important publication factors. However, FP methods became relatively more common over time—although there were no statistically significant trends over time in terms of the specific methods used. While FP models are generally widely implemented in statistical software, their extensions to meta-regression were proposed around the same time as splines.^1^ ^,^ ^7^ It could be that splines have been more widely used in single studies and have therefore remained more familiar to clinical researchers over time.

The more prevalent use of splines does not imply that they are superior. One advantage of splines is their flexibility, allowing researchers to select knots based on *a priori* clinical knowledge.^5^ They can also estimate, in theory, any smooth function. On the other hand, FP models require less input and, therefore, may be less subjective.^3^

The impact factor of papers employing flexible meta-regression methods increased between 2015 and 2021 (Figure 3). This was preceded by a decrease in the period 2011–2014. Fewer studies from the early years of this period were identified and, therefore, the high JIF of the single study from 2011 may skew this trend. Keeping this in mind, the publication impact of reviews using flexible meta-regression generally increased over time.

We found no positive trend between journal impact and reporting quality. However, past research has found evidence for such a trend.^24^ Often, reporting quality is measured with reference to gold-standard checklists, such as PRISMA and MOOSE.^16^ ^,^ ^25^ The lack of trend identified in our study may be due to the lack of such an existing framework for flexible meta-regression to which authors may refer.

We identified other trends in reporting quality. Over two-thirds of reviews had at least one reporting issue. This matches with past research.^8^ ^–^ ^11^ These reviews, which focused on “dose–response” studies, found that reporting was inconsistent. We found that studies using splines had better reporting than those using FP or LR, although this trend was not statistically significant. Despite the lack of statistical significance, this descriptive trend may be surprising, as splines have the additional requirement of having to explicitly specify knots in order to be reproducible.^5^ In the analyzed subsample, splines had better rates of reporting than FP on seven of the eight non-spline-specific items. However, nearly one of five spline studies did not report knot locations. Past studies have also identified issues in reporting knot locations.^5^

There were differences in reporting quality over publication year and number of authors. The most recently published studies (2020–2021) had the lowest reporting quality. Authors should take care to ensure that they clearly and transparently report their use of statistical methods. This is a key component of research reproducibility. Studies with one to three authors appeared to have better reporting than those with four or more. Fleming et al.^26^ examined the qualifications of authors of published systematic reviews and found that the appropriate use of reporting guidelines was associated with the inclusion of methodologists on the team reviewing the research. Similarly, Oltean et al.^27^ found that the reporting of specialized statistical methods, such as those accounting for clustered data, was related to the participation of methodologists on the study team as measured through author self-report. This may be related to the number of authors, although we did not specifically extract this level of detail from included studies. We also found that studies appearing in higher impact journals did not have higher reporting quality than those published in lower impact ones. This may appear counter-intuitive, given past research showing the contrary.^15^ ^–^ ^24^ However, as no guidelines currently exist, editors may not be as demanding on specific reporting norms compared to more well-established fields such as the MA of binary treatments. Given the large number of studies using flexible meta-regression, the development of new reporting guidelines is of clear benefit to both authors and journal editors alike.

### 4.1 Strengths, limitations, and future work

Previous meta-reviews have considered the use and reporting of other methods in systematic reviews, but none to date have considered flexible meta-regression in particular.^8^ ^–^ ^11^ This study provides an insight into how researchers are using these methods in practice and where reporting may be improved in the future.

Our study has certain limitations, however. First, our search may have missed relevant studies.^28^ We conducted a secondary search when it was found that studies employing FP methods were not being included in the initial search. We transparently highlight this search update in this article, although it is a limitation which must be acknowledged. Second, it was not practical to extract data from all N=346 included studies. A random subsample was selected for full extraction. While this is common practice, this is a limitation to the present analysis.^29^ ^–^ ^31^ This could have affected the power of our tests of numerical publication characteristics between the flexible meta-regression method groups. However, the sampled studies were not found to differ from those included in the full sample along available publication characteristics, giving no indication that the subsample is not broadly representative. Third, some of the extracted publication characteristics were subjective in nature (e.g., clinical field). However, they could still be important factors and should be extracted nonetheless.^32^ Fourth, this study was not prospectively registered, although an initial protocol is available on the Open Science Framework (https://osf.io/kwm9p/). The reason for not prospectively registering a protocol is that the scope of the project increased over time. This, however, remains an important limitation. Despite this, we believe the work presented here has merit, particularly in terms of the absence of published reporting guidelines on flexible meta-regression methods. Fifth, given this gap in methodological guidance, we created a new list of reporting items specific to flexible meta-regression. However, these items employed binary yes/no assessments of reporting quality, which could limit their specificity and lead to over-simplified results. Future work could consider elaborating on some of the items and allowing for the evaluation of reporting adequacy and accuracy beyond a simple binary. Sixth, our aim was not to critically appraise the individual flexible meta-regression methods considered in this review. Indeed, each method has its own merits and it is at the discretion of researchers to employ those which best suit their data and research question. Finally, our proposed guidelines were based on an initial review of the included literature as well as our knowledge of the field. However, these guidelines have not been externally validated and were implemented *ad hoc*. Past meta-reviews, however, have used this approach given no previous recommendations.^15^ ^,^ ^32^ We believe that our proposed reporting guidelines provide a foundation for work to develop a validated list of guidelines. Past guidelines, such as PRISMA^16^ and its subject-specific variants,^17^ ^,^ ^18^ have been established through collaborative work between researchers.

## 5 Conclusion

We conducted a systematic search of the clinical literature on flexible meta-regression. We observed an increasing popularity of this method. Splines were the most used method among those considered in this article, with the use of FP growing somewhat over time. Reporting quality was generally poor among all studies, regardless of the method used. Reporting quality did not improve over time. This study may provide a useful guide for those hoping to conduct research in this field. Our findings highlight the need for better reporting among flexible meta-regression studies. This could be aided by the publication of a common set of reporting guidelines, as is the case in other fields of meta-research.

## Data Availability

The extracted data from the subsample of studies included in the review are available on the Open Science Framework (https://osf.io/kwm9p/).

## A Appendix. Summary of selected flexible regression methods

Flexible regression is the application of flexible basis expansions as terms in a regression model. A basis expansion is defined as a set of functions:

(A.1)
$$\begin{array}{r} B(x)=(f_{1}(x),f_{2}(x),\ldots ,f_{m}(x)). \end{array}$$

The basis function is evaluated over the support of *x*. Once a suitable basis is obtained, one may include its elements as predictors in regression models.

For example, one may fit a simple regression model with outcome *y*, as follows:

(A.2)
$$\begin{array}{r} y=\beta_{0}+B(x)\beta +\epsilon , \end{array}$$

where *B* is an n×m matrix of basis vectors, $f_{1},f_{2},\ldots ,f_{m}$ are functions of *x*, $\beta_{0}$ is the linear intercept, $\beta =(\beta_{1},\ldots ,\beta_{m})$ is a vector of length *m* containing the coefficients of the basis vectors, and ϵ is a vector of error terms for the *n* data points.

While simple methods with known disadvantages, such as categorization, polynomial models, and piecewise functions, are sometimes used, recent advances in computing power have made it easy to fit relatively complex expansions.^33^ Select flexible regression models are highlighted below. For a thorough introduction, see Ramsay and Silverman.^34^

### A.1 Polynomial regression

Polynomial regression of degree *m* is based on a basis set of j=1,…,m polynomial transformations $f_{j}$ of *x* as follows:

(A.3)
$$\begin{array}{r} f_{j}(x)=x^{j}. \end{array}$$

These models are a natural extension of simple linear regression. One benefit is that they allow for tailoring the complexity of the trajectory to the desired complexity of the supposed relationship. If one suspects that the true trajectory changes direction exactly once, for instance, it may be sufficient to fit a polynomial model of degree m=2 .^35^ Indeed, by adding more and more polynomial terms of increasing degree into the base set, one may fit trajectories resembling nearly any complex smooth curve.^1^

However, there are some problems with polynomial models. For one, adding bases beyond the quadratic term may lead to erratic fitted trajectories for extreme exposure values.^1^ Further, too many polynomial terms can result in unstable estimates, especially for small sample sizes.^1^ It is also important to ensure that values of x=0 (if any) do not overly influence model estimates.^35^

### A.2 Piecewise functions

A piecewise function is defined based on a partition of the range of *x* into *K* intervals. Within each interval *k*, the function $f_{k}(x)$ takes on a certain value. This value can be constant or based on some transformation of *x*.^36^ The type of piecewise model used depends on what class of transformations of *x* are allowed (e.g., the degree *d* of a polynomial $f_{k}(x)=x+x^{2}+\cdots +x^{d}$ ), the intervals chosen, and whether or not the $f_{k}(x)$ are allowed to jump or are forced to be equal at interval boundaries.

The basis of a piecewise model consists of two parts: the intercept basis and the function basis. The former is simply the basis set of indicator functions for the *K* intervals: $B_{k}^{\text{int}}(x)=I_{k}(x)$ . Here, $I_{k}(x)$ is the indicator function equal to 1 when *x* is within interval *k* and 0 elsewhere. The latter is the basis set of the *K* products of functions $f_{k}(x)$ with their respective interval indicator functions $I_{k}(x)$ : $B_{k}^{\text{fun}}(x)=f_{k}(x)I_{k}(x)$ . This gives the basis set

$$\begin{array}{r} B=\{B_{k}^{\text{int}}{\}}_{k=1,\ldots ,K}\;⋃\;\{B_{k}^{\text{fun}}{\}}_{k=1,\ldots ,K} \end{array}$$

giving us 2K parameters to estimate. Note that it is not necessary to include function bases $B_{k}^{\text{fun}}$ in a piecewise model. In this case, there are K−1 parameters to estimate (excluding the reference level).

One common piecewise model is the continuous piecewise linear model. Here, the $B_{k}^{\text{fun}}$ consist exclusively of linear functions of *x*. Constraints are placed on f(x) to require it to be continuous at each interval boundary $\xi_{k}$ .^36^ This constraint, which forces the limit of the function at each $\xi_{k}$ to exist and be equal to its value at $\xi_{k}$ (i.e., $f(\xi_{k}^{-})=f(\xi_{k})=f(\xi_{k}^{+})$ ), removes parameters from the model by requiring them to be equal. For example, for K=3 , the equality constraint around $\xi_{1}$ gives us $\beta_{1}^{\text{int}}+\xi_{1}\beta_{1}^{\text{fun}}=\beta_{2}^{\text{int}}+\xi_{1}\beta_{2}^{\text{fun}}$ , reducing the number of parameters to estimate by two.

### A.3 Splines

Longitudinal spline models fit a curve over outcome *y* around fixed points (knots) over time *x*. These provide a series of low-order polynomial bases fit to the outcome. This method is very flexible, allowing for a wide range of shapes to be estimated.^36^ Spline methods are implemented in the base R package splines.

To fit a spline model for a trajectory f(x) , one must assume that it can be represented by a linear combination of spline bases with both a fixed degree *d* and knot sequence.^5^ One can then express f(x) as

$$\begin{array}{r} f(x)=\sum_{k=1}^{K+d+1}\beta_{k}B_{k}(x) \end{array}$$

with $B_{k}(x)$ defined as the *k*th element of the basis set B as above on the vector space *V*; $\beta_{k}$ as the associated spline coefficient for $B_{k}(x)$ ; and *K* as the total number of knots. A simple basis spline model such as this leads to d+k+1 free parameters to estimate. Spline models with d=1 (linear splines) fit straight lines between the knots. Models with d=3 (cubic splines) are the standard in flexible modeling as they produce curves which appear smooth to the human eye while limiting the total number of parameters to be estimated.^5^

The main advantage of spline models is that they simplify the complex non-linear estimation of f(x) to a much simpler linear estimation of coefficients $\beta_{k}$ . This lends itself naturally to regression using least-square or ML estimation. However, the necessity of user-defined tuning parameters (degree, knot locations, and number of knots) induces some level of subjectivity to the modeling process.^37^ A general rule-of-thumb is to place knots along *x* with a large amount of data or in locations where one expects f(x) to change rapidly. Subject-level knowledge is therefore useful when determining knot locations.

There are many different types of statistical splines. Examples include B-splines and natural splines. It can be shown that the regression spline models described above are a specific category of continuous piecewise functions.^36^ In this case, the constraints that the overall trajectory f(t) as well as its first and second derivatives are continuous over the range of *x* are added. In general, an order-*m* spline model with knots at $\xi_{1},\ldots ,\xi_{K}$ belongs to the family of piecewise polynomial models of order *m*.^36^

### A.4 Fractional polynomial models

FP models are based on a variable selection algorithm pulled from a limited set of power transformations of *x*.^38^ This method was motivated by the *ad hoc* approaches of early applied statisticians and uses variable selection procedures to determine the best-fitting trajectory.^33^ ^,^ ^39^ Variable selection in FP models is outlined in detail by Royston and Sauerbrei.^33^ This method has been implemented in the R package mfp.^40^

An FP transformation of x>0 is defined as $x^{p}$ with *p* chosen from the set *S* of powers S={−2,−1,−0.5,0,0.5,1,2,3} , where $x^{p}=\text{log}(x)$ for p=0 , by convention. An FP*m* model (that is, an FP model of degree *m*) consists of a basis set B(x) of *m* FP transformations. These are iteratively defined as

$$\begin{array}{r} f_{j}(x)=\left\{ \begin{array}{ll} x^{p_{j}} & \text{ if }p_{j}\neq p_{j-1}\\ f_{j-1}(x)\;log(x) & \text{ else}, \end{array}\right. \end{array}$$

where the basis set is based on j=1,…,m beginning with $f_{0}(x)=1$ and $p_{0}=0$ . The most commonly used degrees of FP models are 1 and 2.^2^ Higher degrees allow for more flexible modeling (i.e., reducing bias in the fitted trajectory), but this comes at the cost of increased model variance, lower parsimony, and increased computational requirements.

In general, FP models are relatively easy to fit and require little user input. They have the ability to estimate a wide range of trajectory shapes.^33^ However, they are limited to strictly positive values of *x*. Timepoints with inherent zeros (e.g., the start of an event) or negative values (e.g., time before or after the event) cannot be used without adjustments. One may add some constant positive value (e.g., the minimum observed value plus one) to correct for this, but this choice is arbitrary. Further, the multiple tests involved in variable selection can lead to overfitting.^41^ Insufficient power to detect a non-linear trend may also arise from the variable selection procedure, particularly for small sample sizes.^33^ Similar to other polynomial regression models, the behavior of the estimated trajectory at the tails of *x* is sensitive to the final transformations selected.^34^

### A.5 Local polynomial regression

Local polynomial regression is a non-parametric regression method which is used to fit smooth curves.^34^ It can be seen as an extension of the moving average and polynomial regression, where low-order polynomials are fit in the local neighborhood of each data point. This method, as opposed to the others summarized above, does not output basis expansion B(x) for use in other regression models, *per se*. Instead, it estimates flexible trajectories using a weighted regression approach.

First, a standard linear model is assumed

$$\begin{array}{r} f(x_{i})=g(x_{i})+\epsilon_{i}, \end{array}$$

where $f(x_{i})$ , i=1,…,n , is the outcome of interest at $x_{i}$ , g(⋅) is an unknown function, and $\epsilon_{i}$ is an error term.

The method below can be generalized to the multivariable case, but, for illustrative purposes, suppose there is a single numeric predictor *x*.^42^ Consider smoothing parameter α>0 . As the user-defined value of α increases, the final fitted trajectory becomes smoother. This parameter defines a window around each data point whereby the fitted curve is weighted such that points nearer to its centre are given more weight than those farther away.^42^ For α≤1 , the neighborhood weight function at point $\left(x_{i},y_{i}\right)$ is

$$\begin{array}{r} w_{i}(x_{i},y_{i})=\left\{ \begin{array}{ll} w(\Delta_{i}(x),\Delta_{\left(q\right)}(x)), & \alpha \leq 1\\ w(\Delta_{i}(x),\Delta_{\left(n\right)}(x)\alpha ), & \alpha >1, \end{array}\right. \end{array}$$

where *w* is some pre-determined weighting function, $\Delta_{i}(x)=|x-x_{i}|$ , $\Delta_{\left(i\right)}(x)$ is its $i^{\text{th}}$ value ordered from smallest to largest along i=1,…,n . A commonly-used weighting function is the tri-cube weight:

$$\begin{array}{r} w(a,b)=\left\{ \begin{array}{ll} (1-(a/b{)}^{3}{)}^{3}, & 0\leq a<b\\ 0, & a\geq b. \end{array}\right. \end{array}$$

The fitted trajectory is then estimated through weighted least-squares regression, with the neighborhood weights used as the model weights. The user can decide on which degree of polynomial, *m*, to fit at each data point. Typically, the highest order considered is a quadratic fit ( m=2 ).

## B Appendix. Full search terms for meta-epidemiological review

See Table 4.

**Table 4:** Ovid MEDLINE search terms Table 4 Long description.

1	(systematic review or meta-analysis).pt.
2	meta-analysis/ or systematic review/ or systematic reviews as topic/ or meta-analysis as topic/ or “meta analysis (topic)”/ or “systematic review (topic)”/ or exp technology assessment, biomedical/ or network meta-analysis/
3	((systematic* adj3 (review* or overview*)) or (methodologic* adj3 (review* or overview*))).ti,ab,kf,kw.
4	((quantitative adj3 (review* or overview* or synthes*)) or (research adj3 (integrati* or overview*))).ti,ab,kf,kw.
5	((integrative adj3 (review* or overview*)) or (collaborative adj3 (review* or overview*)) or (pool* adj3 analy*)).ti,ab,kf,kw.
6	(data synthes* or data extraction* or data abstraction*).ti,ab,kf,kw.
7	(handsearch* or hand search*).ti,ab,kf,kw.
8	(mantel haenszel or peto or der simonian or dersimonian or fixed effect* or latin square*).ti,ab,kf,kw.
9	(met analy* or metanaly* or technology assessment* or HTA or HTAs or technology overview* or technology appraisal*).ti,ab,kf,kw.
10	(meta regression* or metaregression*).ti,ab,kf,kw.
11	(meta-analy* or metaanaly* or systematic review* or biomedical technology assessment* or bio-medical technology assessment*).mp,hw.
12	(medline or cochrane or pubmed or medlars or embase or cinahl).ti,ab,hw.
13	(cochrane or (health adj2 technology assessment) or evidence report).jw.
14	(comparative adj3 (efficacy or effectiveness)).ti,ab,kf,kw.
15	(outcomes research or relative effectiveness).ti,ab,kf,kw.
16	((indirect or indirect treatment or mixed-treatment or bayesian) adj3 comparison*).ti,ab,kf,kw.
17	(multi* adj3 treatment adj3 comparison*).ti,ab,kf,kw.
18	(mixed adj3 treatment adj3 (meta-analy* or metaanaly*)).ti,ab,kf,kw.
19	umbrella review*.ti,ab,kf,kw.
20	(multi* adj2 paramet* adj2 evidence adj2 synthesis).ti,ab,kw,kf.
21	(multiparamet* adj2 evidence adj2 synthesis).ti,ab,kw,kf.
22	(multi-paramet* adj2 evidence adj2 synthesis).ti,ab,kw,kf.
23	or/1-22
24	(“Statistical smoot*” or “Mathematical smoot*” or “Data smoot*” or Smoothing or Smoother or Smoothers or “Bayesian smoot*” or “Computational smoot*” or “Numerical smoot*” or “Parametric smoot*” or “Nonparametric smoot*” or “Non-parametric smoot*”).ti,ab,kw,kf.
25	(spline or splines).ti,ab,kw,kf.
26	(“kernel functio*” or “kernel smoot*” or “kernel metho*” or “kernel regress*”).ti,ab,kw,kf.
27	(“moving averag*” or “moving mean*” or “moving median*” or “moving least-square*” or “moving least square*”).ti,ab,kw,kf.
28	(“rolling averag*” or “rolling mean*” or “rolling median*” or “rolling least-square*” or “rolling least square*”).ti,ab,kw,kf.
29	(“window funct*” or “apodization funct*” or “tapering funct*” or “weight funct*” or “weighting funct*”).ti,ab,kw,kf.
30	(“local regress*” or “local polynomial regress*” or “locally estimated scatterplot smoot*” or “locally-estimated scatterplot smoot*” or “local estimated scatterplot smoot*” or LOESS or LOES or “locally weighted scatterplot smoothing” or “locally-weighted scatterplot smoothing” or “local weighted scatterplot smoothing” or LOWESS or LOWES).ti,ab,kw,kf.
31	(“Digital filte*” or “Savitzky-Golay filte*” or “Chebyshev filte*” or “Elliptic filte*” or “Kalman filte*” or “Kolmogorov–Zurbenko filte*” or “low-pass filte*” or “low pass filte*” or “smoothing filte*”).ti,ab,kw,kf.
32	dynamic or non-linear dynamics or non-linear model or non-linear models or nonlinear dynamic or nonlinear dynamics or nonlinear model or nonlinear models).mp.
33	(“curve fit*” or “line fit*” or “polynomial fit*” or “polynomial regress*” or “fractional poly*” or “fitting line*” or “fitting curve*”).ti,ab,kf,kw.
34	or/24-33
35	23 and 34
36	limit 35 to yr=“2011–2021”

*Note:* The term “fractional poly*” was added to line 33 for the updated search. An anti-join (“NOT” line) was then applied to the results of line 36 for the updated search and those of the initial search to obtain the final results of the updated search.

## C Appendix. Reasons for exclusion at the second stage ( N=73 )

1. Incorrect publication type ( N=54 ).
  - Study is a letter to the editor, conference abstract, conference presentation, protocol, textbook chapter, or other publication which is not a scientific journal article.
2. No flexible meta-regression ( N=7 ).
  - Study does not use at least one flexible meta-regression method.
3. Incorrect scientific or clinical context ( N=5 ).
  - Study is not a biomedical study of human health.
  - Study is an in-vitro study.
  - Study is a brain scan study.
  - Study is solely methodological in nature and does not conduct a real-world data analysis.
4. No meta-analysis ( N=3 ).
  - Study was a systematic review but did not include a quantitative meta-analysis.
5. Study withdrawn ( N=2 ).
  - Study was withdrawn.
6. Study did not use real-world data ( N=1 ).
  - Study employed only simulated or artificial data
7. Not a systematic review ( N=1 ).
  - Study defined itself as a rapid or scoping review rather than a complete systematic review.
  - Study is a pooled analysis of pre-existing datasets not derived from a search.
  - Study is a meta-analysis of studies but studies were selected non-systematically.
  - Study was an analysis from a data consortium.

## Long descriptions

### Table 1 Long description

The table is titled Table 1 Proposed reporting guidelines for flexible meta-regression methods in systematic reviews. It consists of two columns: Reporting item and Item description. The second column begins with the prompt: Did the study…

* Item 1: present any results of the flexible meta-regression method employed?

* Item 2: include a statistical methods section?

* Item 3: report which flexible meta-regression methods were used?

* Item 4: include a citation for the flexible meta-regression methods used?

* Item 5: report which statistical tests were used, if applicable?

* Item 6: clearly state the flexible meta-regression method by name?

* Item 7: report flexible meta-regression method specifically in the methods section?

* Item 8: report details around number and placement of spline knots, if applicable?

* Item 9: report flexible meta-regression method in included figure, if applicable?

Navigate back to Table 1.

### Figure 1 Long description

The flowchart is organized into three vertical phases.

1. Identification Phase.

- Original search: 2,466 records from databases and registers plus 43 from Cochrane. 981 duplicate records were removed.

- Updated search: 59 records from databases and registers plus 0 from Cochrane. 0 duplicate records were removed.

2. Screening Phase.

- Original search: 1,426 records screened, 1,079 excluded. 390 reports sought for retrieval, 0 not retrieved. 390 reports assessed for eligibility, 72 excluded. Reasons for exclusion include out-of-scope publication (n=54), no flexible meta-regression method (n=7), out-of-scope context (n=5), no meta-analysis (n=3), study withdrawn (n=2), and simulated data (n=1).

- Updated search: 59 records screened, 30 excluded. 29 reports sought for retrieval, 0 not retrieved. 29 reports assessed for eligibility, 1 excluded for not being a systematic review.

3. Included Phase.

- Full sample: 318 reports from the original search and 28 from the updated search are combined to reach 346 reports eligible for analysis.

- Subsample: 79 studies were subsampled from the original set and 7 from the updated set.

- Final outcome: The process concludes with 86 studies analyzed.

Navigate back to Figure 1.

### Figure 2 Long description

The X-axis is labeled Publication year and ranges from 2011 to 2021. The Y-axis is labeled Number of studies and ranges from 0 to 50 in increments of 10. Each year features a stacked bar where the total height represents identified studies and a dark bottom segment represents sub-sampled studies.

Data points for identified studies (total height) and sub-sampled studies (dark segment) are as follows:

* 2011: 5 total, 1 sub-sampled.

* 2012: 16 total, 3 sub-sampled.

* 2013: 20 total, 5 sub-sampled.

* 2014: 32 total, 6 sub-sampled.

* 2015: 33 total, 9 sub-sampled.

* 2016: 44 total, 11 sub-sampled.

* 2017: 38 total, 4 sub-sampled.

* 2018: 30 total, 13 sub-sampled.

* 2019: 49 total, 14 sub-sampled.

* 2020: 42 total, 10 sub-sampled.

* 2021: 37 total, 10 sub-sampled.

The trend shows a general increase in identified studies peaking in 2019, while sub-sampled studies fluctuate with a peak in 2019.

Navigate back to Figure 2.

### Table 2 Long description

The table is organized by Publication characteristic in the first two columns and Flexible meta-regression method (n) across five sub-columns: Spline, F P, L R, Other, and Unclear. The final column lists Unique studies (N = 86).

Key data points by category:

* Flexible meta-regression method: Spline is the most common method with 67 unique studies, followed by F P with 17, L R with 5, Other with 2, and Unclear with 2.

* Publication year: The number of studies increased over time, from 9 in 2011-2013 to 27 in 2018-2019 and 20 in 2020-2021.

* Clinical field: Nutrition (37 studies) and Public health (24 studies) are the most frequent fields. Cardiology, Oncology, Nephrology, and Psychology follow with fewer studies.

* Impact factor (J I F): Studies are distributed relatively evenly across Low (29), Mid (29), and High (28) tertiles. The mean J I F for Spline is 4.4, F P is 5.0, and L R is 3.4.

* Number of authors: Most studies have 4 to 6 authors (28) or 7 to 10 authors (26). The mean number of authors is approximately 7.5.

* Number of studies: Most meta-analyses included 11 to 20 studies (28) or 2 to 10 studies (25). The mean number of studies included is 22.8.

* Type of M A: Standard M A accounts for 84 studies, while I P D M A accounts for 2.

* Country: China has the highest representation with 48 studies, followed by the U S A (16) and the U K (11). Other countries like Iran (9) and Italy (5) have smaller counts.

Navigate back to Table 2.

### Figure 3 Long description

The horizontal X axis is labeled Publication year and ranges from 2011 to 2021 in one year increments. The vertical Y axis is labeled Impact factor J I F and ranges from 0 to 20 in increments of 5.

Individual studies are represented by open circles. In 2011, a single outlier appears near 14. From 2012 to 2018, most data points are clustered between 2 and 7. In 2019, 2020, and 2021, the density of points increases, with a notable outlier reaching nearly 20 in 2021.

A dashed black line represents the fitted mean impact factor. It begins at approximately 8 in 2011, follows a downward curve to a trough of roughly 3 in 2015, and then shows a gradual upward trend, ending at approximately 7.5 in 2021. A shaded gray ribbon surrounding this dashed line represents the 95% confidence interval, which is widest at the start in 2011 and narrows as it follows the curve toward 2021.

Navigate back to Figure 3.

### Figure 4 Long description

The horizontal X axis is labeled Publication year, ranging from 2011 to 2021. The vertical Y axis is labeled No. of times method used, with increments of 5 from 0 to 10. A legend at the bottom identifies four categories: Spline (teal), Fractional polynomial (yellow), Local regression (purple), and Other (red).

Data trends by year:

* 2011: 1 Spline.

* 2012: 1 Fractional polynomial, 2 Spline.

* 2013: 1 Fractional polynomial, 4 Spline.

* 2014: 1 Local regression, 1 Fractional polynomial, 4 Spline.

* 2015: 1 Other, 2 Local regression, 2 Fractional polynomial, 6 Spline.

* 2016: 2 Fractional polynomial, 10 Spline.

* 2017: 4 Spline.

* 2018: 12 Spline.

* 2019: 1 Local regression, 2 Fractional polynomial, 11 Spline.

* 2020: 1 Other, 5 Fractional polynomial, 6 Spline.

* 2021: 1 Local regression, 3 Fractional polynomial, 7 Spline.

Overall, the total number of studies increases over time, peaking in 2019. Spline is consistently the largest component of every bar.

Navigate back to Figure 4.

### Figure 5 Long description

A multi-panel figure containing three boxplots. All panels share a common x-axis with three categories: Splines, F P, and L R.

Panel 1 (Left): The y-axis is Impact factor (J I F) ranging from 0 to 20. Splines has a median around 4 with several high outliers up to 19. F P has a higher median near 5 and a wider interquartile range. L R shows a very narrow distribution with a median around 3 and one outlier near 8.

Panel 2 (Center): The y-axis is No. authors ranging from 0 to 20. Splines has a median of 6 with outliers reaching above 20. F P has a higher median around 7 and a tighter box. L R has a median around 6 with a symmetric distribution.

Panel 3 (Right): The y-axis is No. included studies ranging from 0 to 100. Splines has a median around 15 with many outliers extending to 100. F P has a similar median near 18. L R shows the highest median value, approximately 40, with no visible outliers.

Navigate back to Figure 5.

### Table 3 Long description

The table is structured with columns for Reporting items 1 through 9, followed by a summary column titled Any of 1 to 9.

At the top, the aggregate data for Does not meet item shows that 58 out of 86 applicable studies (67.4 percent) failed at least one item. Item 9 had the highest failure rate at 44.4 percent (32 of 72 studies), while Item 1 had the lowest at 1.2 percent.

Under Flexible meta-regression method, failure rates for Any of 1 to 9 are: Spline (59.7 percent), Fractional polynomial (82.4 percent), Local regression (100 percent), Other (50 percent), and Unclear (100 percent).

Under Publication year, the failure rate for Any of 1 to 9 increased over time: 77.8 percent in 2011 to 2013, dipping to 46.7 percent in 2016 to 2017, and rising to 100 percent in 2020 to 2021.

Under Impact factor (J I F), High J I F journals had a 75 percent failure rate for Any of 1 to 9, compared to 62.1 percent for Low J I F and 65.5 percent for Mid J I F.

Under Clinical field, failure rates for Any of 1 to 9 include: Nutrition (64.9 percent), Public health (66.7 percent), Cardiology (77.8 percent), Nephrology (60 percent), Oncology (85.7 percent), Psychology (100 percent), and Other (33.3 percent).

Under Number of authors, failure rates for Any of 1 to 9 range from 43.8 percent (1 to 3 authors) to 75 percent (greater than 10 authors).

Under Number of studies, failure rates for Any of 1 to 9 range from 53.8 percent (21 to 30 studies) to 80 percent (greater than 30 studies).

Under Type of M A, Standard M A had a 67.9 percent failure rate, while I P D M A had a 50 percent failure rate.

Navigate back to Table 3.

### Table 4 Long description

Fallback

Navigate back to Table 4.
